# Alleviation of catabolite repression in *Kluyveromyces marxianus*: the thermotolerant SBK1 mutant simultaneously coferments glucose and xylose

**DOI:** 10.1186/s13068-019-1431-x

**Published:** 2019-04-23

**Authors:** Saet-Byeol Kim, Deok-Ho Kwon, Jae-Bum Park, Suk-Jin Ha

**Affiliations:** Department of Bioengineering and Technology, Kangwon National University, Chuncheon, 24341 Republic of Korea

**Keywords:** Simultaneous cofermentation, Cellulosic biomass, Thermotolerant yeast, *Kluyveromyces marxianus* SBK1

## Abstract

**Background:**

Simultaneous cofermentation of glucose and xylose mixtures would be a cost-effective solution for the conversion of cellulosic biomass to high-value products. However, most yeasts ferment glucose and xylose sequentially due to glucose catabolite repression. A well known thermotolerant yeast, *Kluyveromyces marxianus*, was selected for this work because it possesses cost-effective advantages over *Saccharomyces cerevisiae* for biofuel production from cellulosic biomass.

**Results:**

In the present study, we employed a directed evolutionary approach using 2-deoxyglucose to develop a thermotolerant mutant capable of simultaneous cofermentation of glucose and xylose by alleviating catabolite repression. The selected mutant, *K. marxianus* SBK1, simultaneously cofermented 40 g/L glucose and 28 g/L xylose to produce 23.82 g/L ethanol at 40 °C. This outcome corresponded to a yield of 0.35 g/g and productivity of 0.33 g/L h, representing an 84% and 129% improvement, respectively, over the parental strain. Interestingly, following mutagenesis the overall transcriptome of the glycolysis pathway was highly downregulated in *K. marxianus* SBK1, except for glucokinase-1 (GLK1) which was 21-fold upregulated. Amino acid sequence of GLK1 from *K. marxianus* SBK1 revealed three amino acid mutations which led to more than 22-fold lower enzymatic activity compared to the parental strain.

**Conclusions:**

We herein successfully demonstrated that the cofermentation of a sugar mixture is a promising strategy for the efficient utilization of cellulosic biomass by *K. marxianus* SBK1. Through introduction of additional biosynthetic pathways, *K.* *marxianus* SBK1 could become a chassis-type strain for the production of fuels and chemicals from cellulosic biomass.

**Electronic supplementary material:**

The online version of this article (10.1186/s13068-019-1431-x) contains supplementary material, which is available to authorized users.

## Background

Over the past few decades, research has become more focused on alternative fuel resources due to economic and environmental challenges. Among these resources, biofuels, including ethanol, are the most well known substitutes for liquid transportation fuels [1, 2]. Ethanol production is expected to dramatically increase by the year 2030 [3, 4]. Among the renewable feedstocks for ethanol production, cellulosic biomass has various attractive advantages, such as its abundance and relatively low cost [1, 5, 6]. The main components of cellulosic biomass are cellulose and hemicellulose, which are enzymatically hydrolyzed to glucose and xylose [5, 7]. However, one of the main challenges in glucose and xylose mixed sugar utilization is glucose catabolite repression; a universal mechanism in microorganisms whereby glucose inhibits the consumption of other non-glucose sugars [8, 9]. This is considered to be one of the main hurdles for the efficient conversion of cellulosic biomass hydrolysate to ethanol [10].

In recent years, several engineered *Saccharomyces cerevisiae* strains have been reported to efficiently co-utilize mixed sugars. An engineered *S. cerevisiae* DA24-16BT3 cofermented cellobiose and xylose simultaneously and exhibited improved ethanol yield through overexpression of cellodextrin transporter and β-glucosidase [11]. Kahar et al. demonstrated that mutant *S. cerevisiae* (M2) produced 5.5 g/L ethanol from 20 g/L glucose and 16 g/L xylose with a final yield of 0.15 g/g ethanol [12]. Engineered *S. cerevisiae* SR8#22 produced 26 g/L ethanol from 40 g/L glucose and 40 g/L xylose with a final yield of 0.35 g/g ethanol [13]. An engineered *Kluyveromyces marxianus* over expressed the xylose-specific transporters (GAL2-N376F) and simultaneously cofermented glucose and xylose, producing 50.10 g/L ethanol and 55.88 g/L xylitol [14]. Simultaneous saccharification and fermentation (SSF), or simultaneous saccharification and cofermentation (SSCF) processes are time- and cost-effective strategies often utilized today [15, 16]. In these processes, thermotolerant yeasts are necessary, because saccharification, generally considered as the overall rate-determining step in SSF or SSCF, is performed by cellulase enzymes with high optimal reaction temperatures (45–55 °C) [14, 17]. A well known thermotolerant yeast, *K. marxianus*, is a robust strain used for industrial ethanol production [18] and was previously reported to grow and ferment in environments up to 50 °C [19]. *K. marxianus* also has various benefits over mesophilic yeasts, such as high growth rates, reduced cooling costs, and a broad spectrum of substrates, including xylose [20, 21].

Previously, the *K. marxianus* 17694-DH1 mutant was isolated following a random mutagenesis/directed evolutionary approach and shown to be effective for ethanol production from xylose [22]. However, *K. marxianus* 17694-DH1 displays strong glucose catabolite repression when using glucose and xylose as mixed sugars. In the present study, we employed a directed evolutionary approach using 2-deoxyglucose (2-DG) to overcome catabolite repression in *K.* *marxianus* 17694-DH1. The isolated mutant, *K. marxianus* SBK1, was shown to simultaneously coferment glucose and xylose. Transcriptomic analysis, using RNA-Seq was performed for a deeper understanding of the alleviated catabolite repression.

## Results and discussion

### A directed evolutionary approach for alleviating catabolite repression

In previous studies, 2-DG-resistant mutants demonstrated simultaneous glucose and xylose cofermentation capabilities following relief of glucose catabolite repression [12, 13, 23]. A laboratory-directed evolutionary approach was therefore applied to *K.* *marxianus* 17694-DH1 to alleviate glucose catabolite repression using 2-DG [22]. Pre-cultured cells were initially inoculated into YP media (10 g/L yeast extract, 20 g/L bacto peptone) containing 15 g/L 2-DG and 40 g/L xylose (2-DG/xylose media). When the residual concentration of xylose was almost zero, the cells were transferred into fresh 2-DG/xylose media. The serial sub-cultures were performed for six cycles. The xylose consumption rate during the first cycle was very low (0.10 g/L h), but gradually increased with repeated sub-culturing (see Additional file 1: Fig. S1). The maximum xylose consumption rate was 0.31 g/L h at the fourth enrichment cycle.

To isolate 2-DG-resistant-mutants, cells from the fourth enrichment cycle were plated onto YPX_20_ agar media containing g/L 2-DG (see Additional file 2: Fig. S2). Among 104 colonies grown on the plate, ten large colonies were randomly selected for further testing. Their glucose and xylose cofermentation capabilities were then verified. Interestingly, all ten candidates showed improved cofermentation capabilities compared to the parental strain *K.* *marxianus* 17694-DH1 (see Additional file 3: Fig. S3). The average xylose consumption rates of the ten 2-DG-resistant mutants were more than twofold higher than that of the parental strain. Of those, colony #8 exhibited the highest xylose consumption (0.30 g/L h) and ethanol production rates (0.25 g/L h). Consequently, we selected colony #8 as the best glucose and xylose cofermenting strain and named it *K. marxianus* SBK1.

### Cofermentation capability of *K. marxianus* SBK1

**Fig. 1:**
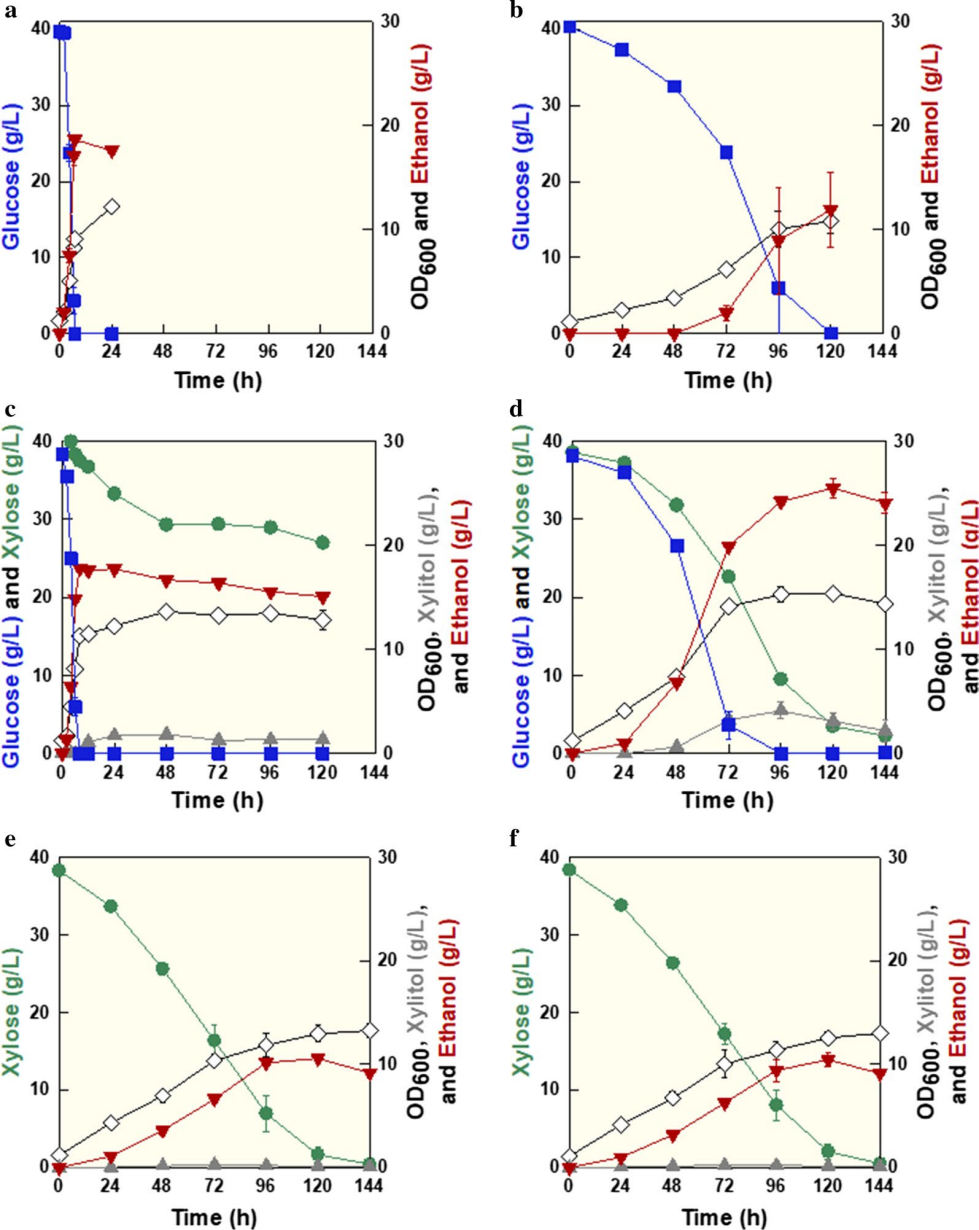
Comparisons of cofermentation performance of the parental strain and *K. marxianus* SBK1 at 30 °C. Glucose fermentation by **a** the parental strain and **b** *K. marxianus* SBK1; glucose–xylose cofermentation by **c** the parental strain and **d** *K. marxianus* SBK1; xylose fermentation by **e** the parental strain and **f** *K. marxianus* SBK1. Symbols: glucose (filled square), xylose (filled circle), OD (open diamond), xylitol (filled upward pointing triangle), and ethanol (filled downward pointing triangle)

To compare fermentation capabilities between the parental and *K. marxianus* SBK1 strains, we performed fermentation experiments in YPD_40_, YPX_40_, or YPD_40_X_40_ media. For glucose fermentation, the parental strain consumed 40 g/L glucose and produced 18.65 g/L ethanol within 7 h (Fig. 1a). The glucose consumption rate, ethanol yield, and productivity were 5.66 g/L h, 0.47 g/g, and 2.66 g/L h, respectively. For xylose fermentation, the parental strain consumed 40 g/L xylose and produced 10.56 g/L ethanol within 120 h (Fig. 1e). The xylose consumption rate, ethanol yield, and productivity were 0.31 g/L h, 0.29 g/g, and 0.09 g/L h, respectively. In the glucose and xylose mixture, the parental strain consumed glucose first, equally as fast as seen for glucose fermentation alone, and thereafter consumed xylose very slowly (only 13.63 g/L xylose was consumed within 120 h) (Fig. 1c). The glucose consumption rate, xylose consumption rate, ethanol yield, and productivity were 4.79 g/L h, 0.11 g/L h, 0.29 g/g, and 0.13 g/L h, respectively. As expected, these results suggest that the parental strain exhibits sequential utilization of the sugar mixture due to glucose catabolite repression.

In the case of *K. marxianus* SBK1, the glucose consumption rate significantly decreased to 0.34 g/L h, whereas the xylose consumption rate was similar to the parental strain over 120 h (Fig. 1e, f). Interestingly, in the glucose and xylose mixture, *K.* *marxianus* SBK1 simultaneously co-consumed the sugar mixture and produced 25.47 g/L ethanol over 120 h (Fig. 1d). Cell growth (OD_600_), ethanol yield, and productivity were 15.36, 0.35 g/g, and 0.21 g/L h, respectively. Ethanol concentration, yield, and productivity increased by 68%, 19%, and 68% compared to the parental strain, respectively. These cofermentation results suggest that glucose catabolite repression might be strongly alleviated in *K.* *marxianus* SBK1, which is consistent with the phenotypes of other 2-DG-resistant mutants [12, 23].

### Verification of catabolite repression in high-glucose concentrations

**Fig. 2:**
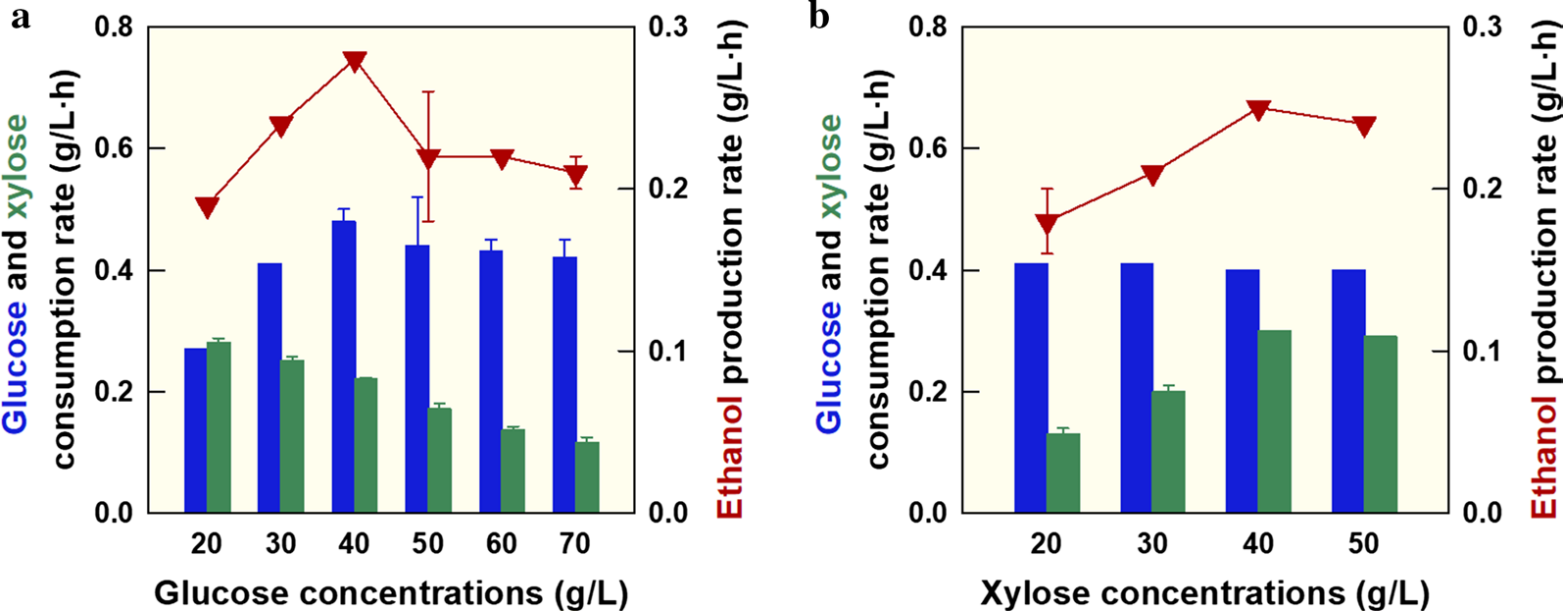
Comparisons of glucose and xylose consumption and ethanol production rates during *K. marxianus* SBK1 cofermentation using increasing glucose or xylose concentrations. **a** Increasing concentrations of glucose (20–70 g/L) with 40 g/L xylose; **b** increasing concentrations of xylose (20–50 g/L) with 40 g/L glucose. Symbols: glucose consumption rate (blue-filled square), xylose consumption rate (green-filled square), and ethanol production rate (filled downward pointing triangle)

*Kluyveromyces marxianus* SBK1 cofermentation experiments were performed with increasing concentrations of glucose and 40 g/L xylose to verify the effect of catabolite repression with high glucose concentrations (see Additional file 4: Fig. S4). When glucose concentrations increased from 20 to 70 g/L, xylose consumption rates gradually decreased from 0.28 to 0.12 g/L h (Fig. 2a). This result suggests that glucose catabolite repression is reduced when glucose concentrations were low; however, catabolite repression persists in *K. marxianus* SBK1 when glucose concentrations were high. As glucose concentrations increased, glucose consumption and ethanol production rates changed in a glucose concentration-dependent manner. Glucose consumption and ethanol production rates increased when the glucose concentration was lower than 40 g/L. However, when the glucose concentration was higher than 40 g/L, glucose consumption and ethanol production rates decreased. Therefore, *K. marxianus* SBK1 exhibited the highest glucose consumption rate (0.48 g/L h) and ethanol production rate (0.28 g/L h) with 40 g/L glucose and 40 g/L xylose.

Xylose concentrations were similarly varied (20–50 g/L), while maintaining 40 g/L glucose constant to investigate the effect of xylose concentration on glucose consumption rates (see Additional file 5: Fig. S5). When xylose concentrations were increased from 20 to 50 g/L, glucose consumption rates remained steady (0.40–0.41 g/L h) (Fig. 2b). Xylose consumption and ethanol production rates gradually increased as xylose concentrations increased. However, with 40 g/L xylose, *K.* *marxianus* SBK1 exhibited the highest xylose consumption (0.30 g/L h) and ethanol production rates (0.25 g/L h). Therefore, subsequent cofermentation experiments were carried out under these optimal substrate concentrations of 40 g/L glucose and 40 g/L xylose.

### Glycolysis pathway transcripts are highly downregulated except for glucokinase-1

To further investigate the nature of alleviated glucose catabolite repression in *K. marxianus* SBK1, quantitative RNA-Seq was performed to compare the parental strain and *K. marxianus* SBK1. Among 1542 altered transcripts, 745 were up- and 797 were downregulated in *K.* *marxianus* SBK1. As *K. marxianus* SBK1 showed reduced glucose consumption rates (Fig. 1b), glycolysis pathway transcripts were analyzed and shown to be downregulated (Table 1). This result suggests the reduced glucose consumption rate of *K. marxianus* SBK1 might be caused by highly downregulated transcripts in the glycolysis pathway. However, glucokinase-1 (GLK1), a glycolysis-initiating enzyme, interestingly showed 20-fold upregulation. Lane et al. reported that catabolite repression can be reduced by modulating the expression of glucose-phosphorylating enzymes, such as GLK1 and hexokinase (HK) [13]. Therefore, *K.* *marxianus* SBK1 *GLK1* and *RAG5*, which encode GLK1 and HK, respectively, were cloned and sequenced using *K. marxianus* NBRC1777 and *K. marxianus* DMKU3-1042 sequencing data as Refs. [24, 25].

**Table 1:** Up- or downregulated transcriptomes in glycolysis pathway by the mutant *K. marxianus* SBK1 compared to the parental strain

	Gene ID	Description	Fold-change	Volume ratio
Upregulation	gene36	Glucokinase-1	20.89	6.03
Downregulation	gene277	Probable phosphoglycerate mutase YOR283W	− 17.60	7.79
	gene2516	Putative phosphoglycerate mutase DET1	− 5.66	5.88
	gene538	Phosphoglycerate kinase	− 4.48	11.79
	gene2788	Fructose-bisphosphate aldolase	− 4.38	12.34
	gene310	6-Phosphofructokinase subunit alpha	− 3.87	8.55
	gene760	Glucose-6-phosphate isomerase	− 3.44	9.36
	gene2517	Triosephosphate isomerase	− 3.40	10.58
	gene3891	Pyruvate kinase	− 3.39	10.79
	gene458	Enolase	− 3.08	13.00
	gene924	Phosphoglycerate mutase 1	− 2.06	12.15
	gene4722	Glyceraldehyde-3-phosphate dehydrogenase 3	− 2.02	12.09

**Fig. 3:**
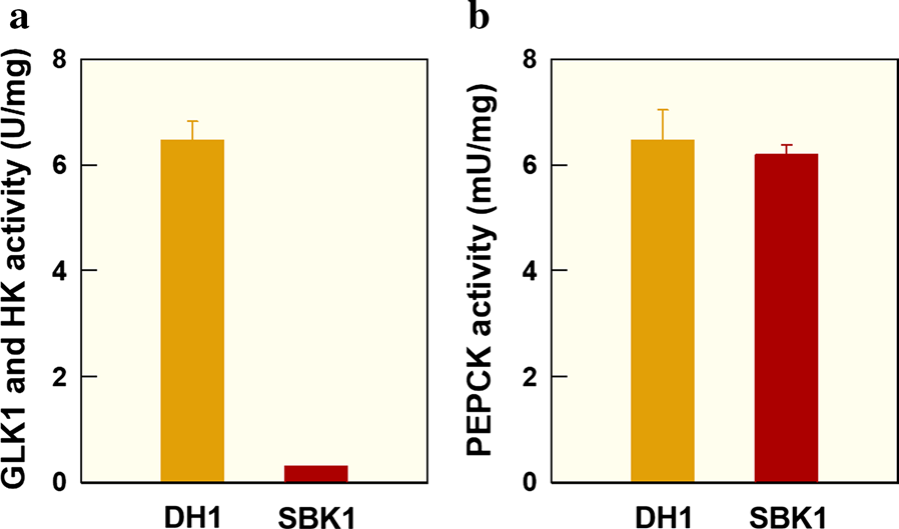
Enzymatic activities of **a** glucokinase-1 (GLK1) and hexokinase (HK); and **b** phosphoenolpyruvate carboxykinase (PEPCK) from the parental strain and *K. marxianus* SBK1. Symbols: *K. marxianus* 17694-DH1 (yellow-filled square), *K. marxianus* SBK1 (red-filled square)

Nucleotide sequences of the respective genes were compared between *K.* *marxianus* SBK1 and the parental strain. Three nucleotide changes in the *K.* *marxianus* SBK1 *GLK1* sequence led to three amino acid changes (see Additional file 6: Table S1), namely the substitution of methionine to isoleucine, serine to proline, and histidine to arginine at positions 13, 301, and 462, respectively. In the *K.* *marxianus* SBK1 *RAG5* nucleotide sequence, adenine was deleted at position 1141, resulting in a frame-shift mutation in the amino acid sequence at position 381. Enzymatic assays were performed to verify enzymatic activity changes of GLK1 and HK between the parental strain and *K. marxianus* SBK1. As shown in Fig. 3a, *K. marxianus* SBK1 GLK1 and HK activities decreased significantly to 0.30 U/mg, compared to 6.47 U/mg in the parental strain. The reduced activities of glycolysis-initiating enzymes are consistent with those observed in other 2-DG-resistant mutants [13, 23]. It was assumed that significantly decreased GLK1 and HK activities might alleviate catabolite repression; therefore, glucokinase-1 was highly upregulated to overcome low GLK1 and HK activities for glucose utilization in *K.* *marxianus* SBK1.

### Verification of key gluconeogenesis pathway enzymes

Gluconeogenesis, the reverse pathway of glycolysis, is generally suppressed by the presence of glucose [26–28]. However, the *K.* *marxianus* SBK1 transcriptomic data indicated phosphoenolpyruvate carboxykinase (PEPCK), a key enzyme in the gluconeogenesis, was highly upregulated (54-fold) [29]. Consequently, the PEPCK gene sequence and enzymatic activity were compared between the parental strain and *K.* *marxianus* SBK1. Six nucleotide changes were identified in the *K.* *marxianus* SBK1 *PCK1* gene (coding for PEPCK) leading to the substitution of serine to threonine and threonine to serine at positions 199 and 386, respectively (Table 2). Four additional nucleotide changes were silent. Since serine and threonine are structurally very similar, it was expected that there would be no significant change in enzymatic activity of PEPCK. As expected, only slight differences were observed in PEPCK enzymatic activities between the parental strain and *K.* *marxianus* SBK1 (Fig. 3b).

**Table 2:** Substitutions of amino acid sequences from key enzymes of glycolysis (GLK1, HK) and gluconeogenesis (PEPCK) pathways

Enzyme	Gene	Substitutions	
		Nucleotide	Amino acid
GLK1	*GLK1*	G39A T901C A1385G	M13I S301P H462R
HK	*RAG5*	C255T A1141Δ	Silence R381fs (frame-shift)
PEPCK	*PCK1*	C75T G489A T595A A921G T1101A A1156T	Silence Silence S199T Silence Silence T386S

### Thermotolerant feature of *K. marxianus* SBK1 for simultaneous cofermentation

**Fig. 4:**
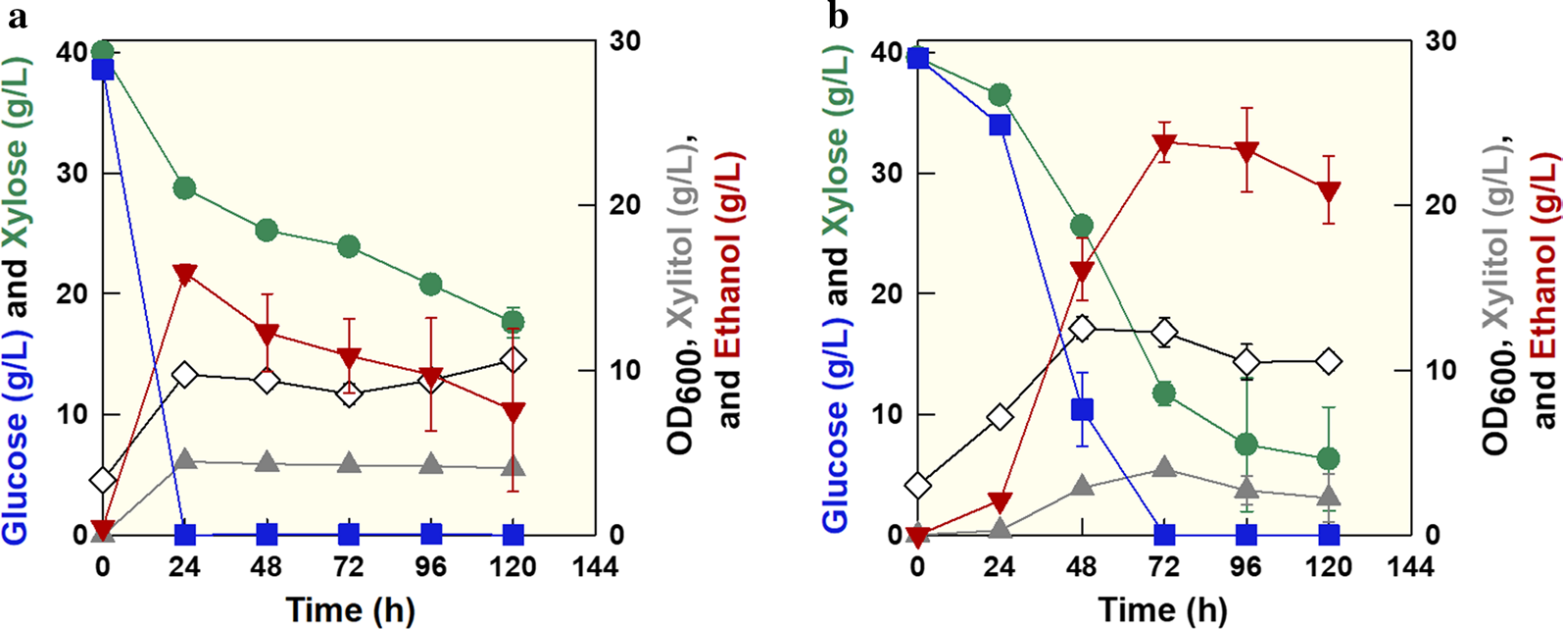
Simultaneous cofermentation of glucose and xylose by **a** the parental strain and **b** *K. marxianus* SBK1 at 40 °C. Symbols: glucose (filled square), xylose (filled circle), OD (open diamond), xylitol (filled upward pointing triangle), and ethanol (filled downward pointing triangle)

For industrial applications of cellulosic biomass utilization using SSF or SSCF, the thermotolerance of *K. marxianus* SBK1 offers various advantages. For evaluation of simultaneous cofermentation of glucose and xylose at high temperature, cofermentation experiments were performed at 40 °C using the parental strain and *K. marxianus* SBK1 (Fig. 4). After consuming 40 g/L glucose and producing 15.89 g/L ethanol within 24 h, the parental strain consumed xylose slowly (Fig. 4a). Glucose and xylose consumption rates were 1.61 g/L h (24 h) and 0.22 g/L h (72 h), respectively. Ethanol yield and productivity were 0.19 g/g and 0.14 g/L h, respectively. These results suggest the parental strain undergoes glucose catabolite repression, even at high cofermentation temperatures. In contrast, *K.* *marxianus* SBK1 showed cofermentation capability at 40 °C (Fig. 4b). Glucose and xylose consumption rates were 0.55 g/L h and 0.39 g/L h over 72 h, respectively. Maximum ethanol concentration, yield, and productivity were 23.82 g/L, 0.35 g/g, and 0.33 g/L h, respectively, following consumption of 40 g/L glucose and 28 g/L xylose. Ethanol yield and productivity improved by 84% and 129% compared with the parental strain. When cofermentation results from 40 °C were compared with those from 30 °C, ethanol concentrations and yields were similar; however, productivity from the 40 °C fermentation was 57% higher than that from the 30 °C fermentation due to higher glucose and xylose consumption rates. This result clearly demonstrates that thermotolerant *K. marxianus* SBK1 efficiently coferments glucose and xylose to ethanol at 40 °C.

## Conclusions

Simultaneous co-utilization of glucose and xylose, the two main components in cellulosic biomass, is an excellent strategy for the efficient production of high-value products. To overcome glucose catabolite repression, we developed the *K. marxianus* SBK1 mutant using a directed evolutionary approach mediated by 2-DG. Following alleviation of glucose catabolite repression, *K. marxianus* SBK1 demonstrated simultaneous cofermentation of glucose and xylose up to 40 °C, resulting in 23.82 g/L ethanol production with a yield of 0.35 g/g and productivity of 0.33 g/L h. Simultaneous cofermentation by the thermotolerant *K. marxianus* SBK1 could offer an efficient strategy for the time- and cost-effective utilization of cellulosic biomass. Further research is required to introduce additional biosynthetic pathways for valuable products using *K.* *marxianus* SBK1.

## Methods

### Strains and media

*Kluyveromyces marxianus* 17694-DH1, capable of fermenting xylose to ethanol, was used as the parental strain in a directed evolutionary approach [22]. For yeast inoculum preparation, pre-culture was carried out in YP media with 20 g/L glucose (YPD_20_) or 20 g/L xylose (YPX_20_). Fermentation experiments were performed using YP media containing YPD_40_, YPX_40_, or YPD_40_X_40_. To investigate the alleviation of catabolite repression, cofermentation experiments were verified using different concentrations of glucose (20–70 g/L) or xylose (20–50 g/L). *Escherichia coli* TOP10 cells (KCTC 22006) were used for gene cloning and sequencing. Each gene was cloned into T-vector using the TOPcloner™ TA core kit (Enzynomics Inc., Korea), which was subsequently used to transform *E. coli* TOP10 competent cells. Transformed *E. coli* TOP10 cells were cultured in LB broth with 50 μg/mL ampicillin.

### A directed evolutionary approach using 2-DG

A directed evolutionary approach was performed using 2-DG to isolate mutants with alleviated glucose catabolite repression. YP media containing 15 g/L 2-DG and 40 g/L xylose (2-DG/xylose media) was inoculated with *K. marxianus* 17694-DH1. When the concentration of xylose was almost zero, cultured cells were transferred to fresh 2-DG/xylose media at OD_600_ 0.10. This process was repeated six times over approximately 50 days. Cells from the fourth transfer, which showed the highest consumption rate of xylose, were used for screening 2-DG-resistant mutants. Cells were harvested and plated on YPX_20_ agar media containing 2-DG at an appropriate dilution. The cofermentation capabilities of ten candidates were evaluated using flask cultivations containing YPD_40_X_40_ media.

### Fermentation experiments

Pre-cultures of *K. marxianus* 17694-DH1 and *K. marxianus* SBK1 were prepared using 5 mL YPD_20_ and YPX_20_, respectively, at 30 °C and 200 rpm. The cells were subsequently inoculated in a 250 mL shaking flask containing 50 mL YPD_40_, YPX_40_, or YPD_40_X_40_ with an initial OD_600_ of 1.00 at 30 °C and 110 rpm. To confirm the effect of catabolite repression by *K. marxianus* SBK1, cofermentation experiments were performed using various concentrations of glucose (20–70 g/L) or xylose (20–50 g/L) at 30 °C. High-temperature cofermentations were carried out with 10 g/L of CaCO_3_ at 40 °C.

### Transcriptomic analysis

Transcriptomic analysis was performed using RNA-Seq to compare expression levels of various transcripts from *K. marxianus* 17694-DH1 and *K. marxianus* SBK1. *K.* *marxianus* 17694-DH1 and *K. marxianus* SBK1 were cultured in YPD_80_ and YPD_40_X_40_ media, respectively. Cultured cells were sampled during the early-exponential phase and total RNA was extracted using the RNeasy^®^ Mini Kit (Qiagen Inc., Germany) after equal adjustment of cell mass. RNA-Seq analysis was performed by a commercial service (Macrogen Inc., Seoul, Korea) using the Illumina Hiseq 4000 sequencer (Illumina Inc., CA, USA). Sequencing data from *K.* *marxianus* NBRC 1777 were used as reference.

### Gene cloning and sequencing

Genomic DNA from *K. marxianus* 17694-DH1 and *K. marxianus* SBK1 was extracted using the i-genomic BYF DNA Extraction Mini Kit (iNtRON Inc., Seongnam, Korea). Target genes encoding glucokinase-1 (*GLK1*), hexokinase (*RAG5*), and phosphoenolpyruvate carboxykinase (*PCK1*) were amplified using Premix Taq™ (Takara Inc., Japan) and the respective PCR primers (see Additional file 7: Table S2). Each amplified gene was purified and cloned into T-vector using the TOPcloner™ TA core kit (Enzynomics Inc., Korea). Gene sequencing was performed by a commercial service (Macrogen Inc., Korea). Based on the resultant data, Lasergene (DNASTAR Inc., WI, USA) was used for sequence analysis.

### Enzymatic assays for hexokinase and phosphoenolpyruvate carboxykinase

For enzymatic assay sample preparation, *K. marxianus* 17694-DH1 and *K. marxianus* SBK1 were cultured in YPX_20_ media. Cells were harvested during the early-exponential growth phase by centrifugation and washed twice with phosphate-buffered saline (PBS) (Noble Bio., Hwaseong, Korea). Washed cells were resuspended in 2 mL assay buffer solution included in each respective enzymatic assay kit, and disrupted using a sonicator (amplitude 40%, pulse on 1 s, and pulse off 1 s for 10 min) on ice. After centrifugation at 4 °C and 18,725×*g* for 5 min, the resulting supernatant was used as the final cell extract for protein quantification and enzymatic assay.

Protein concentrations were determined by the Bradford method, using Bradford reagent (Biosesang Inc., Seongnam, Korea). Based on the results, enzymatic assays were carried out using equally adjusted protein concentrations of the parental and mutant strains. For enzymatic activity analysis, the Hexokinase Colorimetric Assay Kit (Biovision Inc., CA, USA) and Phosphoenolpyruvate Carboxykinase Activity Kit (Biovision Inc.) were used. Enzymatic assay procedures were as per the manufacturer’s instructions.

### Analytical methods

Cell cultures were periodically sampled, and cell densities were measured using optical density (OD) at 600 nm with a GENESYS™ 10S UV–visible spectrophotometer (Thermo Inc., USA). Harvested cells were centrifuged and the concentration of various metabolites (glucose, xylose, xylitol, and ethanol) was analyzed in the resulting supernatant using a high-performance liquid chromatography (HPLC) system (1200 Series, Agilent Inc., USA) with a Rezex ROA-Organic Acid H^+^ column (Phenomenex Inc., USA). The temperature of the column and refractive index detector was maintained at 50 °C. A solution of 0.005 N H_2_SO_4_ was used as the mobile phase at a flow rate of 0.6 mL/min.

## Additional files

**Additional file 1: Fig. S1.** Results of a directed evolutionary approach from the parental strain *K. marxianus* 17694-DH1.

**Additional file 2: Fig. S2.** Comparisons of 2-DG-resistance through spotting assay onto YPX_20_ plates with or without 2-DG.

**Additional file 3: Fig. S3.** Comparisons of xylose consumption rate and ethanol production rate of the selected 2-DG-resistant candidates from glucose and xylose mixture fermentation experiments at 96 h. Symbols: xylose consumption rate (green-filled square) and ethanol production rate (red-filled square).

**Additional file 4: Fig. S4.** Time profiles of cofermentation in mixed culture of glucose and xylose with various concentrations of glucose from 20 g/L to 70 g/L by the mutant *K. marxianus* SBK1. Symbols: glucose (blue-filled square), xylose (green-filled circle), OD (open diamond), xylitol (grey-filled upward pointing triangle), and ethanol (red-filled downward pointing triangle).

**Additional file 5: Fig. S5.** Time profiles of cofermentation in mixed culture of glucose and xylose with various concentrations of xylose from 20 g/L to 50 g/L by the mutant *K. marxianus* SBK1. Symbols: glucose (blue-filled square), xylose (green-filled circle), OD (open diamond), xylitol (grey-filled upward pointing triangle), and ethanol (red-filled downward pointing triangle).

**Additional file 6: Table S1.** Substitutions of amino acid sequences from key enzymes of glycolysis (GLK1, HK) and gluconeogenesis (PEPCK) pathways.

**Additional file 7: Table S2.** Primers used in this study.

## Abbreviations

SSF: Simultaneous saccharification and fermentation
SSCF: Simultaneous saccharification and cofermentation
2-DG: 2-Deoxyglucose
RNA-Seq: RNA-sequencing
YP: Yeast extract-peptone
OD: Optical density
GLK1: Glucokinase-1
HK: Hexokinase
PEPCK: Phosphoenolpyruvate carboxykinase
LB: Luria Broth
PCR: Polymerase chain reaction
PBS: Phosphate-buffered saline
HPLC: High-performance liquid chromatography

## References

[1] Von Sivers M, Zacchi G (1996). Ethanol from lignocellulosics: a review of the economy. Bioresour Technol.

[2] Luo L, Voet E, Huppes G (2009). Life cycle assessment and life cycle costing of bioethanol from sugarcane in Brazil. Renew Sustain Energy Rev.

[3] De La Torre Ugarte DG, English BC, Jensen K (2007). Sixty billion gallons by 2030: economic and agricultural impacts of ethanol and biodiesel expansion. Am J Agric Econ.

[4] Powlson DS, Riche A, Shield I (2005). Biofuels and other approaches for decreasing fossil fuel emissions from agriculture. Ann Appl Biol..

[5] Lee J (1997). Biological conversion of lignocellulosic biomass to ethanol. J Biotechnol.

[6] Ho NW, Chen Z, Brainard AP (1998). Genetically engineered Saccharomycesyeast capable of effective cofermentation of glucose and xylose. Appl Environ Microbiol.

[7] Mosier N, Wyman C, Dale B, Elander R, Lee Y, Holtzapple M, Ladisch M (2005). Features of promising technologies for pretreatment of lignocellulosic biomass. Bioresour Technol.

[8] Kayikci Ömur, Nielsen Jens (2015). Glucose repression inSaccharomyces cerevisiae. FEMS Yeast Research.

[9] Gancedo JM, Gancedo C (1986). Catabolite repression mutants of yeast. FEMS Microbiol Rev.

[10] Rodrussamee N, Lertwattanasakul N, Hirata K, Limtong S, Kosaka T, Yamada M (2011). Growth and ethanol fermentation ability on hexose and pentose sugars and glucose effect under various conditions in thermotolerant yeast *Kluyveromyces marxianus*. Appl Microbiol Biotechnol.

[11] Ha S-J, Galazka JM, Kim SR, Choi J-H, Yang X, Seo J-H, Glass NL, Cate JH, Jin Y-S (2011). Engineered *Saccharomyces cerevisiae* capable of simultaneous cellobiose and xylose fermentation. Proc Natl Acad Sci.

[12] Kahar P, Taku K, Tanaka S (2011). Enhancement of xylose uptake in 2-deoxyglucose tolerant mutant of *Saccharomyces cerevisiae*. J Biosci Bioeng.

[13] Lane S, Xu H, Oh EJ, Kim H, Lesmana A, Jeong D, Zhang G, Tsai C-S, Jin Y-S, Kim SR (2018). Glucose repression can be alleviated by reducing glucose phosphorylation rate in *Saccharomyces cerevisiae*. Sci Rep.

[14] Zhang B, Zhang J, Wang D, Han R, Ding R, Gao X, Sun L, Hong J (2016). Simultaneous fermentation of glucose and xylose at elevated temperatures co-produces ethanol and xylitol through overexpression of a xylose-specific transporter in engineered *Kluyveromyces marxianus*. Bioresour Technol.

[15] Kim J-S, Park J-B, Jang S-W, Ha S-J (2015). Enhanced xylitol production by mutant *Kluyveromyces marxianus* 36907-FMEL1 due to improved xylose reductase activity. Appl Biochem Biotechnol.

[16] Soccol CR, de Souza Vandenberghe LP, Medeiros ABP, Karp SG, Buckeridge M, Ramos LP, Pitarelo AP, Ferreira-Leitão V, Gottschalk LMF, Ferrara MA (2010). Bioethanol from lignocelluloses: status and perspectives in Brazil. Bioresour Technol.

[17] Suriyachai N, Weerasaia K, Laosiripojana N, Champreda V, Unrean P (2013). Optimized simultaneous saccharification and co-fermentation of rice straw for ethanol production by *Saccharomyces cerevisiae* and *Scheffersomyces stipitis* co-culture using design of experiments. Bioresour Technol.

[18] Park J-B, Kim J-S, Jang S-W, Hong E, Ha S-J (2015). The application of thermotolerant yeast *Kluyveromyces marxianus* as a potential industrial workhorse for biofuel production. KSBB J..

[19] Banat IM, Nigam P, Marchant R (1992). Isolation of thermotolerant, fermentative yeasts growing at 52 °C and producing ethanol at 45 °C and 50 °C. World J Microbiol Biotechnol.

[20] Nurcholis M, Murata M, Lertwattanasakul N, Kosaka T, Rodrussamee N, Limtong S, Yamada M (2016). Characteristics of kanMX4-inserted mutants that exhibit 2-deoxyglucose resistance in thermotolerant yeast *Kluyveromyces marxianus*. Open Biotechnol J.

[21] Lertwattanasakul N, Rodrussamee N, Limtong S, Thanonkeo P, Kosaka T, Yamada M (2011). Utilization capability of sucrose, raffinose and inulin and its less-sensitiveness to glucose repression in thermotolerant yeast *Kluyveromyces marxianus* DMKU 3-1042. AMB Express.

[22] Kwon D-H, Park J-B, Hong E, Ha S-J (2018). Ethanol production from xylose is highly increased by the *Kluyveromyces marxianus* mutant 17694-DH1. Bioprocess Biosyst Eng.

[23] Nguyen MT, Lertwattanasakul N, Rodrussamee N, Limtong S, Kosaka T, Yamada M (2015). A *Kluyveromyces marxianus* 2-deoxyglucose-resistant mutant with enhanced activity of xylose utilization. Int Microbiol..

[24] Inokuma K, Ishii J, Hara KY, Mochizuki M, Hasunuma T, Kondo A (2015). Complete genome sequence of *Kluyveromyces marxianus* NBRC1777, a nonconventional thermotolerant yeast. Genome Announc..

[25] Lertwattanasakul N, Kosaka T, Hosoyama A, Suzuki Y, Rodrussamee N, Matsutani M, Murata M, Fujimoto N, Tsuchikane K, Limtong S (2015). Genetic basis of the highly efficient yeast *Kluyveromyces marxianus*: complete genome sequence and transcriptome analyses. Biotechnol Biofuels.

[26] Rolland F, Winderickx J, Thevelein JM (2002). Glucose-sensing and-signalling mechanisms in yeast. FEMS Yeast Res.

[27] Klein CJ, Olsson L, Nielsen J (1998). Glucose control in *Saccharomyces cerevisiae*: the role of Mig1 in metabolic functions. Microbiology.

[28] Hers H, Hue L (1983). Gluconeogenesis and related aspects of glycolysis. Ann Rev Biochem.

[29] Hedges D, Proft M, Entian K-D (1995). CAT8, a new zinc cluster-encoding gene necessary for derepression of gluconeogenic enzymes in the yeast *Saccharomyces cerevisiae*. Mol Cell Biol.

