# Supplementary material for: Alleviation of catabolite repression in Kluyveromyces marxianus: the thermotolerant SBK1 mutant simultaneously coferments glucose and xylose
Source: Biotechnol Biofuels. 2019 Apr 23;12:90. doi: 10.1186/s13068-019-1431-x (PMC6477723; doi:10.1186/s13068-019-1431-x)

**Additional file 1**

**Fig. S1.** Results of a directed evolutionary approach from the parental strain *K. marxianus* 17694-DH1.


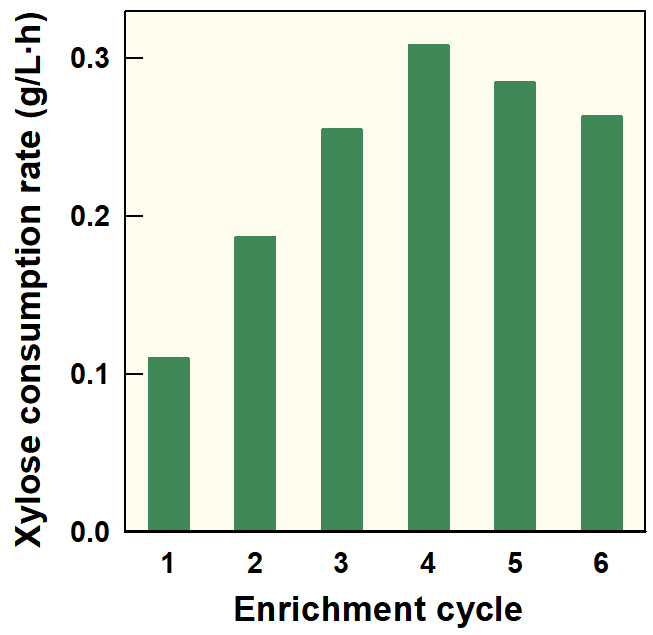

Supplement: Supplementary file 1 — Additional file 1: Fig. S1. Results of a directed evolutionary approach from the parental strain K. marxianus 17694-DH1. [file 13068_2019_1431_MOESM1_ESM.docx]
