# Supplementary material for: Alleviation of catabolite repression in Kluyveromyces marxianus: the thermotolerant SBK1 mutant simultaneously coferments glucose and xylose
Source: Biotechnol Biofuels. 2019 Apr 23;12:90. doi: 10.1186/s13068-019-1431-x (PMC6477723; doi:10.1186/s13068-019-1431-x)

**Additional file 2**

**Fig. S2.** Comparisons of 2-DG-resistance through spotting assay onto YPX_20_ plates with or without 2-DG.


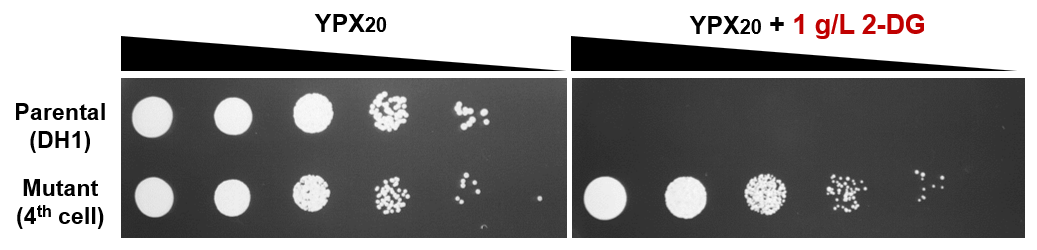

Supplement: Supplementary file 2 — Additional file 2: Fig. S2. Comparisons of 2-DG-resistance through spotting assay onto YPX20 plates with or without 2-DG. [file 13068_2019_1431_MOESM2_ESM.docx]
