# Supplementary material for: Alleviation of catabolite repression in Kluyveromyces marxianus: the thermotolerant SBK1 mutant simultaneously coferments glucose and xylose
Source: Biotechnol Biofuels. 2019 Apr 23;12:90. doi: 10.1186/s13068-019-1431-x (PMC6477723; doi:10.1186/s13068-019-1431-x)

**Additional file 3**

**Fig. S3.** Comparisons of xylose consumption rate and ethanol production rate of the selected 2-DG-resistant candidates from glucose and xylose mixture fermentation experiments at 96 h. Symbols: xylose consumption rate (■) and ethanol production rate (■).


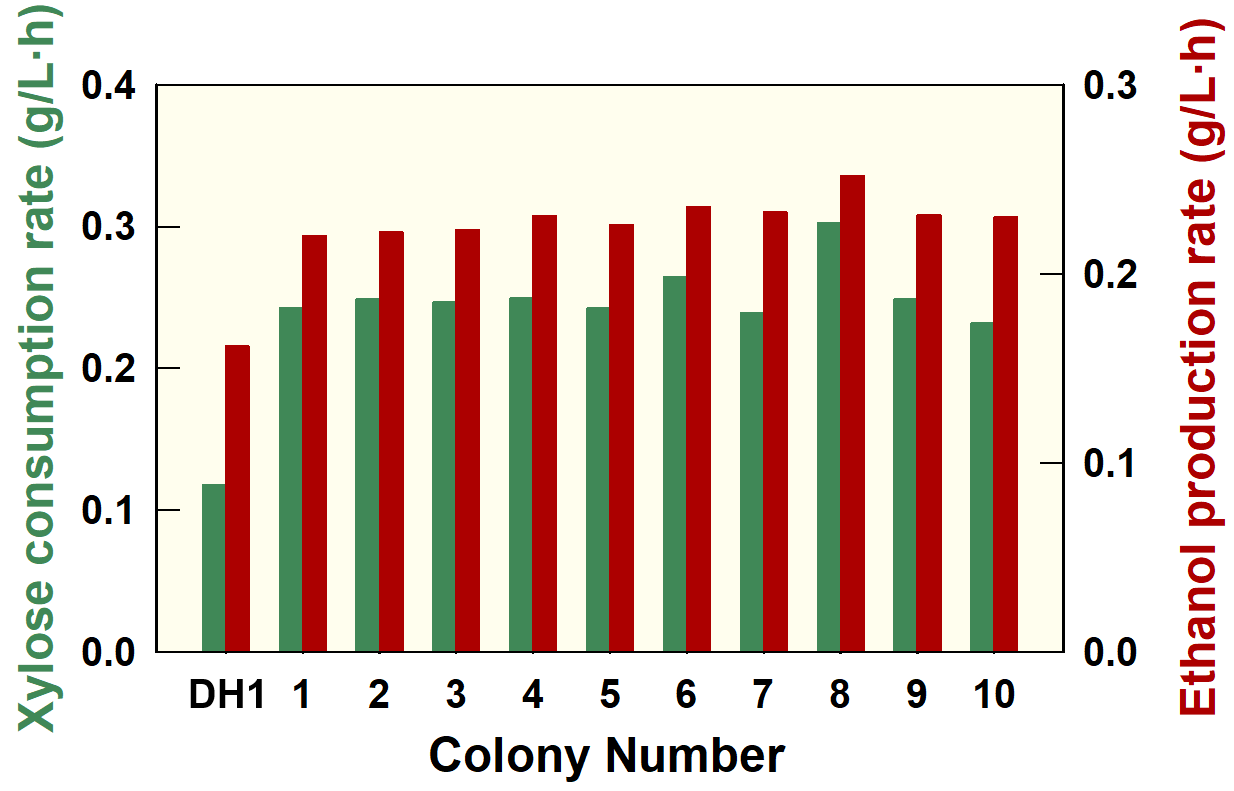

Supplement: Supplementary file 3 — Additional file 3: Fig. S3. Comparisons of xylose consumption rate and ethanol production rate of the selected 2-DG-resistant candidates from glucose and xylose mixture fermentation experiments at 96 h. Symbols: xylose consumption rate (green-filled square) and ethanol production rate (red-filled square). [file 13068_2019_1431_MOESM3_ESM.docx]
