# Supplementary material for: Alleviation of catabolite repression in Kluyveromyces marxianus: the thermotolerant SBK1 mutant simultaneously coferments glucose and xylose
Source: Biotechnol Biofuels. 2019 Apr 23;12:90. doi: 10.1186/s13068-019-1431-x (PMC6477723; doi:10.1186/s13068-019-1431-x)

**Additional file 4**

**Fig. S4.** Time profiles of cofermentation in mixed culture of glucose and xylose with various concentrations of glucose from 20 g/L to 70 g/L by the mutant *K. marxianus* SBK1. Symbols: glucose (■), xylose (●), OD (◇), xylitol (▲), and ethanol (▼).


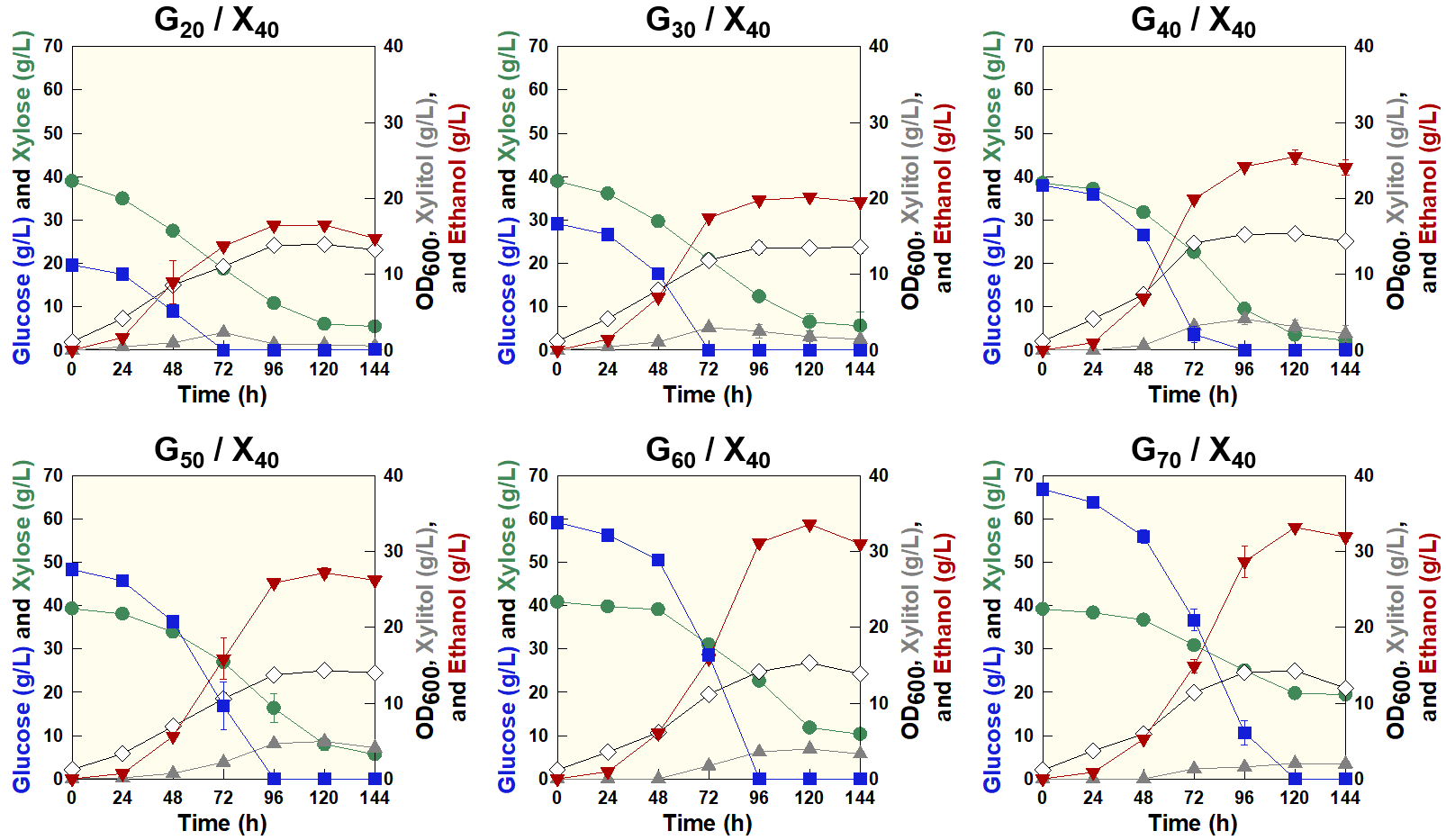

Supplement: Supplementary file 4 — Additional file 4: Fig. S4. Time profiles of cofermentation in mixed culture of glucose and xylose with various concentrations of glucose from 20 g/L to 70 g/L by the mutant K. marxianus SBK1. Symbols: glucose (blue-filled square), xylose (green-filled circle), OD (open diamond), xylitol (grey-filled upward pointing triangle), and ethanol (red-filled downward pointing triangle). [file 13068_2019_1431_MOESM4_ESM.docx]
