# Supplementary material for: Alleviation of catabolite repression in Kluyveromyces marxianus: the thermotolerant SBK1 mutant simultaneously coferments glucose and xylose
Source: Biotechnol Biofuels. 2019 Apr 23;12:90. doi: 10.1186/s13068-019-1431-x (PMC6477723; doi:10.1186/s13068-019-1431-x)
Supplement: Supplementary file 6 — Additional file 6: Table S1. Substitutions of amino acid sequences from key enzymes of glycolysis (GLK1, HK) and gluconeogenesis (PEPCK) pathways. [file 13068_2019_1431_MOESM6_ESM.docx]

**Additional file 6**

**Table S1.** Substitutions of amino acid sequences from key enzymes of glycolysis (GLK1, HK) and gluconeogenesis (PEPCK) pathways.

| Enzyme | Gene | Substitutions | |
| --- | --- | --- | --- |
|  |  | Nucleotide | Amino acid |
| GLK1 | *GLK1* | G39A  T901C  A1385G | M13I  S301P  H462R |
| HK | *RAG5* | C255T  A1141Δ | Silence  R381fs (frame-shift) |
| PEPCK | *PCK1* | C75T  G489A  T595A  A921G  T1101A  A1156T | Silence  Silence  S199T  Silence  Silence  T386S |
