# Supplementary material for: Alleviation of catabolite repression in Kluyveromyces marxianus: the thermotolerant SBK1 mutant simultaneously coferments glucose and xylose
Source: Biotechnol Biofuels. 2019 Apr 23;12:90. doi: 10.1186/s13068-019-1431-x (PMC6477723; doi:10.1186/s13068-019-1431-x)
Supplement: Supplementary file 7 — Additional file 7: Table S2. Primers used in this study. [file 13068_2019_1431_MOESM7_ESM.docx]

**Additional file 7**

**Table S2.** Primers used in this study.

| Primer names | Sequence (5'→3') |
| --- | --- |
| *GLK1*_Fw | ATGTCAGACCCTAAATTAACAAAAGC |
| *GLK1*_Rv | TTAGTAGTCGGCTGTTAAAGCAC |
| *RAG5*_Fw | ATGGTTCATTTAGGTCCAAAGAAGC |
| *RAG5*_Rv | TTAAGCACCTTCGATACCAACAGA |
| *PCK1*_Fw | ATGTCCCCAAGCAAAATTCACCAC |
| *PCK1*_Rv | TTATAGTTGAGGACCAGCAGCCAAAA |
